# No-boundary thinking for artificial intelligence in bioinformatics and education

**DOI:** 10.3389/fbinf.2023.1332902

**Published:** 2024-01-08

**Authors:** Prajay Patel, Nisha Pillai, Inimary Toby

**Affiliations:** ^1^ Chemistry Department, University of Dallas, Irving, TX, United States; ^2^ Department of Computer Science, Mississippi State University, Starkville, MS, United States; ^3^ Biology Department, University of Dallas, Irving, TX, United States

**Keywords:** bioinformatics and computational biology, artificial intelligence, ChatGPT, education -active learning, no-boundary thinking, NIBLSE

## Abstract

No-boundary thinking enables the scientific community to reflect in a thoughtful manner and discover new opportunities, create innovative solutions, and break through barriers that might have otherwise constrained their progress. This concept encourages thinking without being confined by traditional rules, limitations, or established norms, and a mindset that is not limited by previous work, leading to fresh perspectives and innovative outcomes. So, where do we see the field of artificial intelligence (AI) in bioinformatics going in the next 30 years? That was the theme of a “No-Boundary Thinking” Session as part of the Mid-South Computational Bioinformatics Society’s (MCBIOS) 19th annual meeting in Irving, Texas. This session addressed various areas of AI in an open discussion and raised some perspectives on how popular tools like ChatGPT can be integrated into bioinformatics, communicating with scientists in different fields to properly utilize the potential of these algorithms, and how to continue educational outreach to further interest of data science and informatics to the next-generation of scientists.

## 1 Introduction

The emergence of computational biology in the 1960s can be attributed to three pivotal technological and conceptual developments (Hagen, 2000). First, the expanding repository of protein and amino acid sequences (Chang et al., 1965) provided new avenues for quantitative analysis but required computational power to derive insights from these early datasets (Levitt, 2001). Second, molecular biology’s central idea that macromolecules, such as DNA and RNA, contain biological information was developed, establishing a theoretical framework for the use of computation to interpret genetic information (Crick, 1958). Lastly, high-speed digital computers, developed during World War II for the purpose of breaking codes, have now made these once scarce machines more widely accessible to biologists (Luscombe et al., 2001). Though not yet universally adopted, this newfound computing power gave initial adopters the ability to analyze biological data and develop foundational algorithms. Together, these factors provided key data resources, a conceptual basis, and essential computing tools that allowed pioneers to blend computation with biology’s most pressing questions. Paulien Hogeweg and Ben Hesper coined the term “bioinformatics” in 1970, referring to the study of information processes in biotic systems (Hesper and Hogeweg, 1970). At an early stage in computational biology and bioinformatics, Margaret Dayhoff contributed significantly to the development of the field, and her Point Accepted Mutation matrix (PAM) quantified evolutionary changes in amino acids based on protein sequences (Dayhoff, 1972). Los Alamos National Laboratory was one of the first institutions to analyze the increasing amount of nucleic acid sequence data (Williams et al., 1978). By the end of the 1970s, these early efforts laid the foundation for bioinformatics to emerge as a quantitative approach to molecular biology.

Over the 1980s, bioinformatics continued to develop as a vital interdisciplinary field, playing a pivotal role in advancing molecular biology. The establishment of nucleic acid and protein sequence databases, such as GenBank (NCBI, 1982) at the National Institutes of Health in 1982, was a significant milestone during this period. Meanwhile, ambitious efforts to sequence the human genome (National Research Council, 1988) and the development of computational tools for assembling and analyzing these large datasets began. A decade after its introduction, bioinformatics became an essential component of research in molecular biology and biotechnology. Through the 1990s, high-throughput sequencing marked an era of rapid growth and innovation in bioinformatics. Projects such as the Human Genome Project (International Human Genome Sequencing Consortium, 2004) have driven the necessity for algorithms and databases to store, organize, and analyze massive amounts of genomic data. To address these challenges, bioinformaticians have developed new sequence alignment tools, molecular modeling techniques, and approaches to identifying genes and regulatory regions in DNA with degree programs established by the mid-1990s to train scientists in this rapidly evolving field. A landmark achievement of bioinformatics was the completion of the Human Genome Project in 2003. Bioinformaticians have developed advanced algorithms for statistical and machine learning (ML) analysis to gain insight from this flood of omics data. Cloud computing enabled the storage and analysis of large genomic datasets. By the late 2000s, bioinformatics had become an integral part of a wide range of fields, including molecular biology and drug discovery such that data-driven biology empowered by bioinformatics became the defining feature of the 21st century.

Researchers are developing artificial intelligence (AI) and other computational tools at a rapid pace due to the availability of data generated in the big data era. Popular AI tools like ChatGPT stand to serve as a starting point for the next-generation of AI models that can assist in research in bioinformatics. As part of the 19th annual meeting for the Mid-South Computational Bioinformatics Society (MCBIOS), attendees participated in a no-boundary discussion to discuss how bioinformatics will evolve in the next 30 years. The discussion centered around four major themes: educating the next-generation, leveraging AI tools, gaining new research perspectives, and engaging students and faculty in outreach.

## 2 Directions

### 2.1 Educating the next generation

Bioinformatics education plays a pivotal role in training the next-generation of scientists to oversee the deluge of biological data in the modern era. As bioinformatics continues to evolve, the need for core competencies in this field becomes increasingly important for both students and researchers.

Previously, the NSF-funded group, Network for Integrating Bioinformatics into Life Sciences Education (NIBLSE) published core competencies for education in life sciences (Sayres et al., 2018). These core competencies in bioinformatics provide students with a solid foundation, enabling them to adapt to the rapidly changing landscape of biological data analysis. These competencies serve as a roadmap for students, ensuring they gain the essential skills needed to address complex biological questions. The foundation of the core competencies encompass proficiency in programming, statistical analysis, database management, and biological knowledge. These competencies go beyond technical skills, also involving critical thinking, problem-solving, and effective communication. Bioinformatics core competencies encourage interdisciplinary collaboration as students learn to bridge the gap between biology and computational sciences, and enable researchers to work seamlessly across various scientific domains, fostering innovation and discoveries. Integrating core competencies into bioinformatics curricula ensures that students receive a well-rounded education.

Adopting competency-based learning outcomes empowers educators to tailor programs to meet specific research needs. Teaching bioinformatics core competencies can be challenging due to the rapid evolution of technologies and tools. Instructors must stay current and adapt their teaching methods to keep pace with the field. A sturdy foundation in core competencies is essential for researchers to leverage the full potential of bioinformatics in their work. Researchers proficient in these competencies can expedite data analysis, drive hypothesis-driven research, and enhance the reproducibility of their studies. Advancements in AI and ML are redefining bioinformatics core competencies, necessitating a continuous learning approach. The integration of omics data and single-cell sequencing presents new challenges and opportunities in bioinformatics education. Bioinformatics education and the cultivation of core competencies are vital for driving advancements in the life sciences. As the field of bioinformatics continues to grow, educators and researchers must adapt, ensuring that core competencies remain relevant and robust. To meet the evolving demands of bioinformatics, educational institutions should establish continuous professional development programs for instructors. Aggregating resources for bioinformatics education into central repositories has been a viable avenue to make instructional content more readily available (Dinsdale et al., 2015). Additionally, as part of the efforts by the NIBLSE community to address the training of students, they conducted a survey of 1,260 faculty across the US to identify their thoughts on what such training should involve. Approximately 95% of those surveyed agreed with the statement, “I think Bioinformatics should be integrated into undergraduate life sciences education.” Nevertheless, there exist variations in faculty viewpoints across diverse types of institutions. These discoveries offer valuable understanding regarding diverse educational outlooks and the challenges educational institutions might encounter while assimilating bioinformatics into their life sciences programs. It is crucial to acknowledge the disparities between the educational requirements of bioinformaticians and life scientists, as well as the distinctions between the objectives of undergraduate education compared to graduate or professional education. The prevailing discourse in the literature concerning bioinformatics education has primarily centered around the training of bioinformaticians or the advancement of graduate and professional skills, neglecting the context of undergraduate education. As the popularity of data science and AI tools increases, the tools we use for education need to be more inclusive and target students who may not have programming experience but are interested in bioinformatics. Several tools are starting to emerge as interactive demonstrations to utilize in active learning environments.

Two examples of NIBLSE tools featured during the 2023 MCBIOS workshop delivered by Drs. Elizabeth Ryder, Adam Kleinschmit, and William Morgan were: 1) An inquiry based and “under the hood” approach for incorporating molecular sequence alignment in introductory undergraduate biology courses; and 2) RNAseq data analysis using Galaxy. Both resources exemplify valuable, freely accessible tools that educators can employ in their classrooms, utilizing them effectively for bioinformatics education.

### 2.2 Leveraging artificial intelligence tools in bioinformatics

With tools and platforms like ChatGPT, DALL-E, and other language/image neural network models currently serving as the zeitgeist for AI research, research workflows incorporate AI tools to increase knowledge of the chemical space in cheminformatics and various genomes in bioinformatics. The rise of generative pre-trained transformer (GPT) models—ChatGPT—has led to a boom in research highlighting ChatGPT in both a positive and negative light. Applied as a tool for glaucoma patients, ChatGPT provided generic and repetitive information that is written at a level to be understood by those at a higher grade level (12.5 ± 1.6) in comparison to the explanations provided on AAO.org (9.4 ± 3.5) according to the Flesch-Kincaid Readability Test (Wu et al., 2023). In chemical education, ChatGPT was implemented in the laboratory to show how that would affect students’ ability to write lab reports (Humphry and Fuller, 2023; West et al., 2023). These studies found that students still need to develop the skills to effectively ask ChatGPT the right questions if the goal is for ChatGPT to be an effective substitute for writing lab reports even though ChatGPT cannot successfully generate both specific experimental details and meaningful data analysis. However, ChatGPT can be used as a curation model to scan the literature effectively and essentially create a computer-based lab assistant through prompt engineering. In work by Zheng et al., 26,257 parameters of roughly 800 metal organic frameworks (MOFs) sourced from peer-reviewed studies were used to train a GPT model to predict MOF crystallization conditions with over 87% accuracy and answer questions about chemical reactions and synthesis procedures from a data-grounded perspective (Zheng et al., 2023). Therefore, with proper training of both model and user, popular AI models like ChatGPT can benefit scientists in both an educational and research environment.

Pertaining to bioinformatics, Vision Transformers (ViT) models (Dosovitskiy et al., 2020) like AlphaFold (Jumper et al., 2021) have dramatically improved protein structure prediction from amino acid sequences, enabling better understanding of protein function. Transformers are also instrumental in providing highly improved performance and reducing complexity in the segmentation of medical images (Deng et al., 2021). Language-Image models such as CLIP (Contrastive Language-Image Pre-Training) (Radford et al., 2021) are gaining popularity in bioinformatics for integrating images and text. One application is generating textual descriptions of the contents and biological context visible in microscopy images through bioimage captioning (Aono et al., 2023). More broadly, CLIP provides a way to connect multimodal biomedical data by associating images and text from papers, reports, and social media to gain new biological insights through multi-modal biomedicine (Lin et al., 2023). By learning visual concepts from natural language supervision, CLIP offers an efficient framework for making sense of diverse image and text data in biology. Recently, domain-specific pre-trained models (Najgebauer et al., 2020) have leveraged the strengths of large-scale language models while ensuring the language representations are finely tuned for unique challenges and opportunities in each domain. BioBERT (Lee et al., 2020) is a prime example demonstrating the effectiveness of pre-training language models on biomedical text corpora (Black et al., 2022), which creates representations specialized for biomedical natural language processing tasks. Similarly, BioMegatron (Shin et al., 2021) was pre-trained on massive biomedical datasets totaling 18 billion words. These datasets encompassed materials such as PubMed abstracts, clinical notes, and full-text articles from the biomedical domain, which exposed the model to a vast vocabulary within the biomedical space. The pre-trained BioMegatron model can then be fine-tuned and applied to various downstream tasks like question answering, natural language inference, and PICO (Population, Intervention, Comparison, Outcome) extraction. These models highlight how bioinformatics research successfully leverages AI models.

### 2.3 Gaining new perspectives

As scientists sequestered in our respective fields, we often network and collaborate with people that closely align with our research in the experimental “wet” fields. For those who straddle the boundaries of self-taught skills in coding within their respective field—biology, chemistry, physics—reaching out to colleagues in different departments, like computer science, can benefit both parties by providing insight into new research avenues. For example, when deciding which ML models to use, people often do not take the time to understand the nuances of each method. As is the case for various unsupervised machine learning techniques for clustering, the distribution shape of data plays a significant role in whether the data can be effectively clustered through centroid-based (K-means), density-based (DBSCAN), or distribution-based clustering (Gaussian Mixture Models). If one is not careful with the type of clustering technique chosen, the data interpretation could lead to an ineffective conclusion. Therefore, a better understanding of the mathematical and coding principles used to create these models serves the community at large to better understand the tools for AI model development rather than blindly trusting that the models are rigorous.

However, collaborating with experimental scientists in one’s respective field can still lead to gaining a new perspective. Focusing on rational drug design, computational efforts include neural networks built on physical, structural, and chemical properties used to screen potential drug candidates. Collaborative efforts between computational and experimental researchers can lead to insight into the chemical nature of various drug-like molecules. For example, discussions with an experimental chemist has led to insight into how the subtle structural effects such as the orientation of the lone pairs on the N atoms in the N,N-disubstituted piperazine ring moiety of drug molecules like fluphenazine and trazodone leads to favored solubility of a drug molecule in different solvents, which affects how drugs are absorbed in the human body Draper et al. (2023). These subtle features may get overlooked by a data scientist focused on quantitative accuracy of AI models and this anecdote serves as a reminder when training AI models. Overall, discussions with colleagues in different fields can help one link computer-generated data to the underlying scientific principles that dictate the training of AI models.

### 2.4 Student and faculty engagement and outreach

As part of the opportunities to address barriers to implementation of bioinformatics core competencies into curriculum, NIBLSE developed the idea of “incubators” in collaboration with the Quantitative Undergraduate Biology Education and Synthesis (QUBES; https://qubeshub.org/) network. The goal of these small online faculty groups is to develop new curricular modules and nurture a growing network of faculty implementing bioinformatics modules in the classroom (Ryder et al., 2020). An incubator is a short (6–10 weeks), focused, online community that refines an existing teaching lesson submitted by its author into a more polished and widely useable learning resource (Ryder et al., 2020). Incubator participants are composed of both experts and novices on the resource topic to ensure both accuracy and accessibility of the finished product. Active NIBLSE members are recruited to participate in an incubator as well. The incubators themselves can provide useful support networks; faculty that participate in the incubator are also likely to implement the resource in their classrooms.

As faculty, we need to continue to refine our approach to content delivery for students. One example is teaching through demonstrations—conducting mini-experiments and analyzing data during live demonstrations in the classroom. Incorporating real-world examples and/or case studies are increasingly becoming essential so that students can develop an awareness of past, current, and future perspectives in the field. Instructors should continue to challenge students to share their opinions, by encouraging brainstorming sessions, group projects, and oral presentations. Students should leave the classroom knowing what it means to be a good researcher and collaborator, as this is often the case with research-related jobs and professions where the work is conducted as part of a team of investigators.

The ability to read the primary literature is crucial in training students to think like scientists. For upper-level courses or graduate programs, coursework should continue to reflect dedicated exercises where students are expected to spend time reading and analyzing primary research journal articles. The importance of digging deeper should be emphasized to help reinforce the students’ understanding of primary literature and help them improve scientific writing skills. Practical applications need to be highlighted for the theories being discussed. Students learn concepts, but most importantly, the ability to troubleshoot, actualize, and practice use case scenarios. The goal is to provide the students with a depth of understanding that helps further their understanding of the subject area. Learning is a distinct mechanism by which there is a harmonious exchange of information from the delivery process to the application of the shared information.

As informatics continues to experience technological advances, faculty should remain abreast of emerging developments and adaptable to integrate specialized skills training into courses. Some examples of these skills in the current Bioinformatics and Machine Learning for Biology courses at the University of Dallas (UD) include creating interactive visualizations, managing data analysis workflows, contributing to data reproducibility and transparency, and data parsing and interpretation. These technical skills have broad interests from students and offer them important insights. Another pathway for implementing the core competencies is through Course-based Undergraduate Research Experiences (CUREs), which splits students into teams to undertake an active research project over the course of a semester under the guidance of the instructor. Project updates in the form of reports and presentations build the students’ practical research skills and can lead to student-led published work. At UD, the Physical Chemistry II lab is a CURE that explores how computational chemistry can apply to a research topic that interests the student, e.g., computational drug discovery. Computational CUREs work as low-cost course-based research that addresses elements of the core competencies previously published by NIBLSE.

## 3 Conclusion

Overall, this No-Boundary Thinking perspective covers the numerous areas of growth we see for utilizing AI tools in research and in the classroom. Collaborative instructional tools like the core competencies and course-based research experiences can help train the next-generation to promote the active development of AI models for continued growth in bioinformatics research.

## Data Availability

The original contributions presented in the study are included in the article/Supplementary Material, further inquiries can be directed to the corresponding authors.
